# Progestin and adipoQ receptor 7 (PAQR7) mediate the anti-apoptotic effect of P4 on human granulosa cells and its deficiency reduces ovarian function in female mice

**DOI:** 10.1186/s13048-024-01348-w

**Published:** 2024-02-06

**Authors:** Jia Li, Yiting Liu, Jinxia He, Zixuan Wu, Fang Wang, Jian Huang, Liping Zheng, Tao Luo

**Affiliations:** 1 School of Basic Medical science, Nanchang University, Nanchang, Jiangxi 330031 China; 2 Key Laboratory of Reproductive Physiology and Pathology of Jiangxi Province, Nanchang University, Nanchang, Jiangxi 330031 China; 3 Institute of Life Science and School of Life Science, Nanchang University, Nanchang, Jiangxi 330031 China; 4 Reproductive Medical Center, Jiangxi Maternal and Child Health Hospital, Affiliated Maternal and Child Health Hospital of Nanchang University, Nanchang, Jiangxi 330006 China; 5 School of Public Health, Nanchang University, Nanchang, Jiangxi 330006 China; 6 Jiangxi Provincial Key Laboratory of Preventive Medicine, Nanchang University, Nanchang, 330006 P.R. China

**Keywords:** Apoptosis, Granulosa cell, Ovarian function, Progesterone, Progestin and adipoQ receptor 7(PAQR7)

## Abstract

**Purpose:**

PAQR7 plays a key role in cell apoptosis as a progesterone membrane receptor. The physiological mechanism of PAQR7 in ovarian function and its anti-apoptotic action in mammals remain poorly understood.

**Methods:**

We first added 0.2 µM aminoglutethimide (AG), an inhibitor of endogenous progesterone (P4) secretion, and transfected si*PAQR7* co-incubated with P4 in human KGN cells to identify granulosa cell apoptosis, respectively. Additionally, we used Paqr7 knockout (PAQR7 KO) mice to assess the role of PAQR7 in the ovary.

**Results:**

The PAQR7 deficiency significantly increased apoptosis of KGN cells, and this significant difference disappeared following P4 supplementation. The *Paqr7*^−/−^ female mice showed a prolonged estrous cycle, reduced follicular growth, increased the number of atresia follicles, and decreased the concentrations of E2 and AMH. The litters, litter sizes, and spontaneous ovulation in the *Paqr*7^−/−^ mice were significantly decreased compared with the *Paqr*7^+/+^ mice. In addition, we also found low expression of PAQR7 in GCs from human follicular fluids of patients diagnosed with decreased ovarian reserve (DOR) and ovaries of mice with a DOR-like phenotype, respectively.

**Conclusions:**

The present study has identified that PAQR7 is involved in mouse ovarian function and fertilization potential. One possible mechanism is mediating the anti-apoptotic effect of P4 on GC apoptosis via the BCL-2/BAX/CASPASE-3 signaling pathway. The mechanism underlying the effect of PAQR7 on ovarian development and aging remains to be identified.

**Supplementary Information:**

The online version contains supplementary material available at 10.1186/s13048-024-01348-w.

## Introduction

Granulosa cells (GCs) play a crucial role in follicular development and the maintenance of ovarian function [1]. Dysregulation of genes associated with GCs function and apoptosis can result in ovarian insufficiency [2, 3]. Previous studies have demonstrated that females with diminished ovarian reserve (DOR) exhibit significantly increased apoptosis of GCs compared to those with normal ovarian function, and this heightened level of GC apoptosis is more likely to be linked to adverse outcomes in in vitro fertilization (IVF)/intracytoplasmic sperm injection procedures [4–8], which is one of the main causes of female infertility, greatly threatening women’s reproductive health.

Progesterone (PROG, P4) was among the earliest identified hormones and is an endogenous 21-carbon steroid hormone synthesized from cholesterol via pregnenolone. It serves as a crucial gonadal hormone synthesized in the corpus luteum of the ovaries and also by the placenta during pregnancy [9, 10]. Progesterone can act directly on granulosa cells of mice, rats, and women via genomic or nongenomic signalling pathways to slow the rate of mitosis and preserve granulosa cell viability [9, 11–13]. The non-genomic effects of P4 are generally rapid and have been observed in rat granulosa cells through the activation of a wide variety of signal transduction pathways, including mitogen-activated protein kinase, Ca^2+^, protein kinase G [14–16].

Progestin and adipoQ receptor 7 (PAQR7), also called membrane P4 receptor α (mPRα), is a membrane protein with seven transmembrane domains, similar to G protein-coupled receptors [17, 18]. Recent studies have shown that PAQR7 has important physiological functions in a variety of reproductive tissues [19, 20]. The PAQR7 is an intermediary in progestin induction of oocyte maturation and stimulation of sperm hypermotility in fish [21, 22]. In mammals, Kowalik et al. reported that the highest *Paqr7* mRNA expression was found on days 11–16 and 17–20 of the estrous cycle compared with other stages of the estrous cycle and pregnancy in the bovine uterus, suggesting that PAQR7 has been implicated in progesterone regulation of uterine function [23, 24]. In addition, Sueldo et al. reported that PAQR7 participates in the antimitotic action of P4 in human GCs and luteal cells, and P4’s ability to suppress entry into the cell cycle was dependent on PAQR7 but not nuclear progesterone receptor (PGR) [25]. These studies demonstrated that PAQR7 could mediate multiple progestin functions during the female reproductive cycle, and P4’s ability to regulate human follicle development is dependent, in part, on the expression levels of PAQR7. However, the physiological mechanism of PAQR7 in human GCs by mediating the anti-apoptotic action of P4 remains unclear, and whether PAQR7 is related to ovarian function is still unknown.

In this study, we clarified the role of PAQR7 in mediating the anti-apoptotic action of P4 and downstream apoptosis pathways by knocking down PAQR7 in human KGN cells. Furthermore, we used a *PAQR7*-knockout (*Paqr7*^-/-^) mouse to investigate the function and mechanism of PAQR7, in order to obtain insight into its association with ovarian function. Finally, we examined the PAQR7 protein levels in GCs isolated from the follicular fluids of patients with DOR and in the ovaries of mice with a DOR-like phenotype. Therefore, our study may shed new light on delaying the occurrence of ovarian aging.

## Materials and methods

### Animals

All animal procedures were conducted in accordance with the recommendations of the Animal Centre of Nanchang University guidelines and approved by the Animal Care and Use Committee of Nanchang University (Permit Number: SYXK2021-0004, Supplementary Tables S4). Mice were housed 2–4 per cage, had free access to water and food and were maintained at 20–25 °C with a 12 h light and 12 h dark photoperiod.

A mouse model exhibiting a DOR-like phenotype was constructed according to our previous report [26]. Six-week-old C57BL/6J mice (wild type) were treated with Tripterygium wilfordii polycoride tablets (TPT, De Ende Pharmaceutical Co. LTD, Zhejiang, China) by infusing the stomachs of the mice. Briefly, we used a lavage needle to gently administer the TPT liquid (0.6 mg/(100 g/day) for 9 days) by inserting the needle into the mice’s mouths, reaching the esophagus. The control group received saline using the same method ( Fig. 7).

A *Paqr7*-knockout mouse (*Paqr7*^-/-^), C57BL/6J strain, was constructed using the clustered regularly interspaced short palindromic repeats (CRISPR)-CRISPR associated protein 9 (Cas9) method employing the gRNA pair (gRNA1: 5’-TCCAGAAAAGTGGCGGCACCTGG-3’; gRNA2: 5’-ACGAGCCTCTGCACGCCCGTTGG-3’). The identification of F2 offspring in the ovaries of *Paqr7*^*-/-*^ female mice was performed through PCR using genotyping primers (GP-F: 5’-CAGCGTGACTCCTTGATCTTCAAACTC-3’; GP-R: 5’-GCAGGCCCAAGGATTGGCTAG-3’) and Western blot assay. Two-month-old female mice were used for the subsequent experiments (Supplementary Fig. S2).

### Cell culture and treatments

The human granulosa cell line KGN was purchased from Shanghai Hongshun Biotechnology Co. KGN cells were cultured in (DMEM)/F-12 medium (Corning) supplemented with 10% fetal bovine serum (FBS, Gibco) and a penicillin-streptomycin-gentamicin additive. They were maintained in a 5% CO_2_ cell incubator at a constant temperature of 37 °C.

The day before transfection, 2 × 10^5^ human GCs (the KGN cell line) were seeded in 6-well plates at 60–70% confluence. KGN cells were cultured in vitro with charcoal/dextran-treated serum and incubated with 0.2 µM aminoglutethimide (AG; HY-B0237, MCE, Shanghai, China), an inhibitor of endogenous P4 secretion [14], 1 µM P4 (P0130, Sigma-Aldrich, St. Louis, MO, USA) or 0.2 µM AG plus 1 µM P4 for 24 h at 37 °C.

To knock down *PAQR7*, the KGN cells were cultured in vitro and transfected with PAQR7 siRNA (genOFFTM st-h-PAQR7_003:GTCCTGTGGTGCATCGTAT) or siRNA-negative (control) for 48 h according to the manufacturer’s instructions (SIGS0016288-1, Ribobio, Guangzhou, China). The day before transfection, 2 × 10^5^ KGN cells were seeded in 6-well plates at 60–70% confluence. The complete culture medium was replaced with a solution containing 2 mL of siRNA-negative or siPAQR7 with 1 µM P4. After 12 h, the siRNA and P4 solution was replaced with complete culture medium and the KGN cells were cultured for 48 h.

### Collection of human follicular fluids

Human follicular fluids were obtained from females with DOR (*n* = 11) and females with normal ovarian storage function group (NOR, *n* = 11); they had undergone IVF treatment in the reproductive medical centre at Jiangxi Provincial Maternal and Child Health Hospital (Nanchang, Jiangxi, China) between October 2020 and October 2021 (Supplementary Tables S1 and S2). This study was approved by the ethics committee of Jiangxi Provincial Maternal and Child Health Hospital. Written informed consent was signed by the participants or their relatives. Subjects were classified into the DOR and normal groups according to the Bologna criteria and the ESHRE guidelines [27, 28]. The process involved a comprehensive consideration of the various definitions of DOR [29, 30] to formulate a standardized set of DOR criteria (Supplementary Table S1).

### Apoptosis

To determine the apoptosis of KGN cells, an Annexin V Probe Kit (Beyotime, Beijing, China) was used according to the manufacturer’s instructions. Briefly, KGN cells were incubated with a mixture of Annexin V-APC, PI, and Annexin V Binding Buffer in a ratio of 1:1:100 at 37 °C for 30 min in the dark [31, 32]. After washing, stained cells were fixed and co-stained with 4′,6-diamidino-2-phenylindole (DAPI) (C0065-50, Solarbio, Beijing, China). Fluorescent signals (red and green) were examined with an Olympus IX73 microscope.

### Western blot analysis

GCs were isolated from human follicular fluids according to Ferraretti et al. [27, 33]. . Total protein was extracted from GCs in human follicular fluids, mouse ovarian tissue and KGN cells using radio immunoprecipitation assay (RIPA) buffer (R0010, Solarbio, Beijing, China) containing 1× protease inhibitor cocktail (HY-K0010, MedChemexpress). Total protein (30 µg) was separated with sodium dodecyl sulphate–polyacrylamide gel electrophoresis (with a 12% gel) and transferred onto polyvinylidene difluoride membranes (IPVH00010, Sigma-Aldrich, St. Louis, MO, USA). Then, the membranes were incubated with the appropriate primary antibodies: anti-PAQR7 (named: mPRａ,1:750, bs-17741R, Bioss, Beijing, China), anti-Bcl2 (1:1000, 26593-1-AP, Proteintech, Wuhan, China), anti-Bax (1:5000, 50599-2-Ig, Proteintech, Wuhan, China), anti-cleaved-Caspase-3 (1:2000, Cell Signaling Technology (#9664), MA, USA), and anti-GAPDH (1:5000, 10494-1-AP, Proteintech, Wuhan, China) for 14 h at 4 °C. Next, they were incubated with horseradish peroxidase (HRP)-conjugated goat anti-rabbit IgG (1:5000, SA00001-2, Proteintech, Wuhan, China) or goat anti-mouse IgG (1:5000, SA00001-1, Proteintech, Wuhan, China). The protein bands were visualised using the enhanced chemiluminescence detection kit (S6009M, Uelandy, Shanghai, China). Analysis of the relative level of the target proteins was conducted by quantifying the gray value of target bands detected by the corresponding antibodies normalized to those detected by the anti-GAPDH antibody using Image J software (version 1.44, National Institutes of Health, Bethesda, MD, USA). Each experiment was repeated at least three times.

### Real-time quantitative PCR (RT-qPCR)

Total RNA was extracted from GCs in human follicular fluids, mouse ovarian tissues and KGN cells using the TRIzol reagent (Invitrogen, Waltham, MA, USA) in accordance with the manufacturer’s instructions. Then, 1 µg of RNA was reverse-transcribed to complementary DNA (cDNA) (RR047A, PrimeScript™ RT reagent Kit, TaKaRa, Kusatsu, Japan) and quantified with the SYBR Green reagent (RR430A, TaKaRa, Kusatsu, Japan) in a CFX96 real-time PCR detection system (Bio-Rad, Hercules, CA, USA) [34]. The 2^−ΔΔCt^ method was used to calculate the relative expression. The transcript levels of the examined genes were normalized to the *GAPDH* transcript level. The gene-specific primers for RT-qPCR are shown in Table S3.

### Definition of the phase of estrous cycle

To determine the phase of the estrous cycle, we collected vaginal discharge smears and stained them with hematoxylin and eosin (HE). Macroscopic and microscopic analyses of the ovaries were performed, and these were categorized as being in the follicular phase. The estrous cycle is divided into proestrus, estrus, metestrus and diestrus. We collected smears continuously for 12 days, covering 2–3 cycles.

### The fertility rate of Paqr 7^−/−^ female mice

We selected sexually mature mice with different genotypes (male mice typically 2–3 months, and female mice typically 3 months). Each pair consisted of one male and one female were housed in individual cages; The cage pairings were as follows: wild-type female mouse with wild-type male mouse (+/+♂X +/+♀), wild-type male mice and *Paqr7*^−/−^ female (+/+♂ X -/-♀). We continuously observed their fertility over a period of approximately six months, recording both the timing and number of each litter born.

### The spontaneous ovulation

Five 2-month wild-type (*Paqr7*^+/+^) female mice and five *Paqr7*^−/−^ female mice were each paired with adult males known to be fertile. The cages were closed overnight, and the presence of vaginal plugs in female mice was checked the following morning. The fallopian tube of female mice with the vaginal plugs were promptly dissected and placed in M2 culture solution containing 0.5 mg/mL hyaluronidase to clean the oocytes. Subsequently, a statistical count was conducted.

### Histological analysis of ovarian tissue and ovarian follicle counts

We examined the 2-month-old wild-type (*Paqr7*^+/+^) female mice (*n* = 5) and 2-month-old *Paqr7*^−/−^ female mice (*n* = 7). In brief, after fixation in 4% paraformaldehyde solution, the ovaries were dehydrated in ethanol and xylene, and embedded in paraffin. Paraffin-embedded ovaries were serially sectioned at 5 μm thickness using a microtome (RM2255, Leica, Berlin, Germany). Then, the ovarian sections were dewaxed in xylene, rehydrated in ethanol and stained with hematoxylin and eosin (HE) [35]. All sections were then examined uisng an Olympus IX73 microscope, and ovarian follicles were counted following established protocols [36, 37].

### The index of ovary

The mice were sacrificed, and the ovaries were removed under aseptic conditions and cleaned in a PBS solution. The ovaries were weighed after removing the surface fluid with an absorbent paper. The ovary index was calculated using the following formula: Ovary index (‰) = ovary weight / body weight * 1000 [38].

### Measurement of hormone levels

We collected serum samples from 2-month-old mice via retroorbital plexus at diestrus or proestrus stages of the estrous cycle. Additionally, we collected blood samples from the saphenous vein and the tail vein in mice to assess the levels of follicle-stimulating hormone (FSH) (E-EL-M0511c, Elabscience, China), oestradiol (E2) (E-EL-0150c, Elabscience, China), progesterone (P4), and anti-Müllerian hormone (AMH) (E-EL-M3015, Elabscience, China) using the corresponding enzyme-linked immunosorbent assays, following to the manufacturer’s instructions. In addition, the age and stages of the estrous cycle at the time of serum collection for hormone levels (experiments in line 237) were specified.

### Terminal deoxynucleotidyl transferase mediated dUTP-biotin nick end labelling (TUNEL) assay

The apoptotic rates of ovarian sections were detected using the one-step TUNEL in situ apoptosis kit, following the manufacturer’s instructions (E-CK-A320, Elabscience, China). After exposure to the TUNEL reaction mixture for 1 h, the sections were washed twice with phosphate-buffered saline (PBS) for 10 min and then counterstained with 4’,6-diamidino-2-phenylindole (DAPI) (C0065-50, Solarbio, Beijing, China) for 5 min [39]. Subsequently, the slices were examined using an Olympus IX73 microscope and the apoptotic rate was analyzed using IPP 6.0 Software.

### Reactive oxygen species (ROS) assay

The level of intracellular ROS was determined by dihydroethidium staining using the ROS assay Kit (D7008, Sigma-Aldrich, St. Louis, MO, USA). Briefly, *PAQR7*^−/−^ ovarian sections were washed twice with PBS and subsequently incubated with ROS dye solution for 30 min at 37 °C. The reaction mixture was then replaced with PBS. The sections were examined using an Olympus IX73 microscope.

### Statistical methods

All analyses were performed using GraphPad Prism 5.01 (GraphPad Software, Inc., San Diego, CA, USA). The data are expressed as the mean ± standard error of the mean (SEM), and all data were analyzed by normality test and ANOVA before comparison. Analyses of independent sample t-tests were used for the comparison between the treated and control groups. *P* < 0.05 was considered significant; *P* < 0.01 and *P* < 0.001 were considered extremely significant.

## Results

### P4 inhibited the apoptosis of human KGN cells

**Fig. 1:**
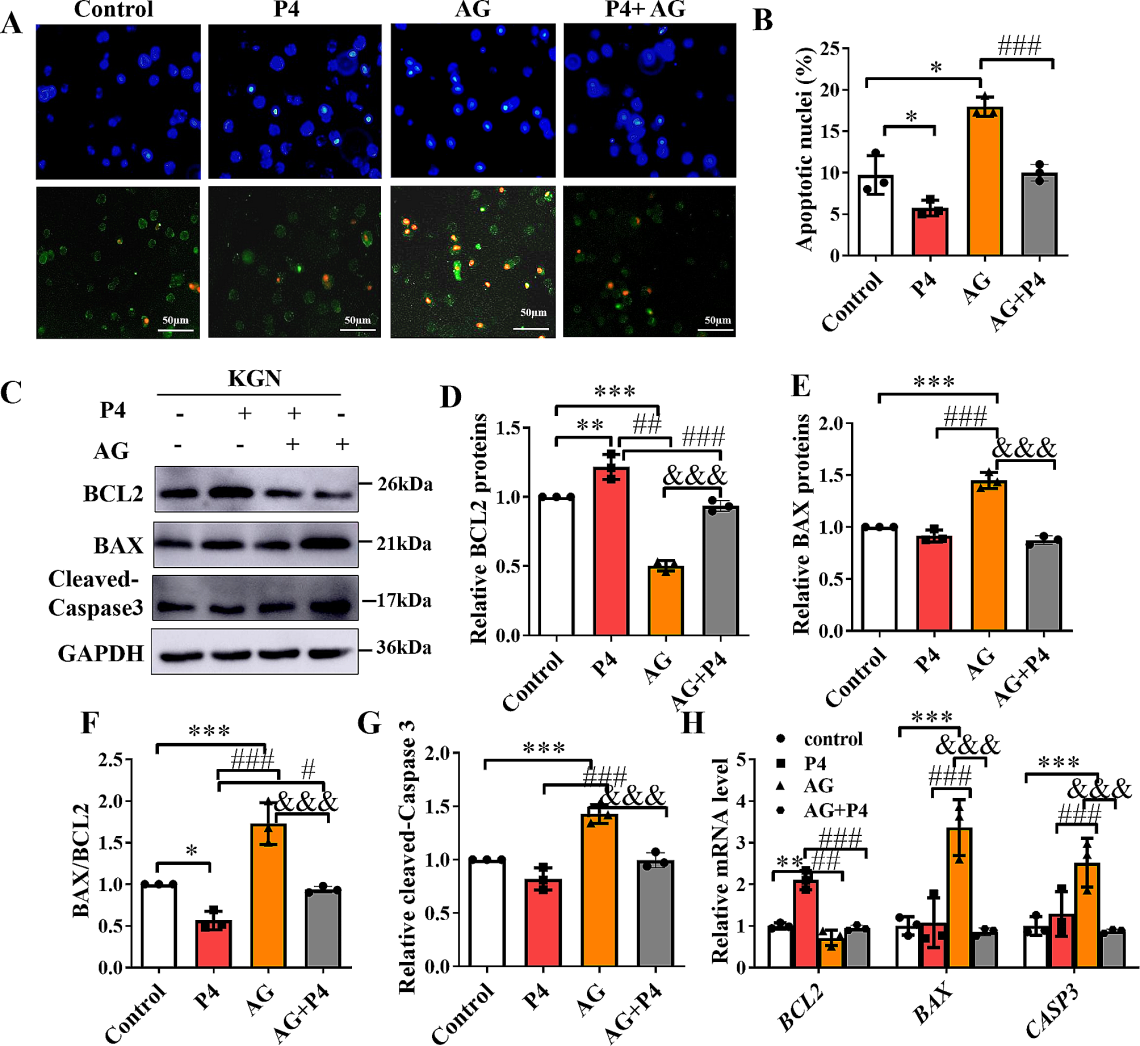
Effects of progesterone (P4) on apoptosis of human KGN cells. (**A** and **B**) Effects of P4 on the apoptosis of KGN cells was examined by annexin V-Fluorescein 5-isothiocyanate/propidium iodide staining. Early apoptotic cells show green fluorescence (Annexin V+/PI-), while late apoptotic cells and necrotic cells show green and red fluorescence (Annexin V+/PI+). 4′,6-diamidino-2-phenylindole (blue) was used to stain the nuclei. Scale bars represent 50 μm. (**C**) A sample of western blot analysis measuring the protein levels of Cleaved-CASPASE-3-3, BAX and BCL2 in KGN cells after P4 treatment or aminoglutethimide (AG), or P4 and AG co-incubation (P4 + AG). (**D**-**G**) Statistical analyses of BCL2 (**D**), BAX (**E**), BAX/BCL2 ratio (**F**) and Cleaved- CASPASE-3 (**G**) were conducted by quantifying the gray value of target bands normalized to that detected by the anti-GAPDH antibody (loading control) using Image J software. (**H**) The mRNA levels of apoptosis-related genes (*CASPASE*-3, *BAX* and *BCL2*) in KGN cells were analyzed after P4 treatment or AG, P4 and AG co-incubation (P4 + AG) by real-time quantitative PCR. Values are presented as the mean ± SD. The experiment was repeated three times. *P < 0.05, One-way ANOVA analysis

When KGN cells were incubated with P4 (P4 group) only, both early apoptosis (green in Fig. 1A) and late apoptosis (red in Fig. 1A) in KGN cells decreased (Fig. 1B). Blocking the secretion of endogenous P4 by treating cells with aminoglutethimide (AG) resulted in an increase in the apoptosis of KGN cells. When exogenous P4 was added, it inhibited the AG-induced apoptosis increase (Fig. 1A and B). The levels of cleaved CASPASE-3 and BAX expressions were much higher, and the BCL2 expression level was lower in the AG group compared with the control and P4 groups (Fig. 1C, and Fig. 1D-E, G). In addition, the BAX/BCL2 ratio showed the highest in the AG group (Fig. 1F). AG exposure also changed the *CASPASE-3*, *BAX*, and *BCL2* mRNA levels in KGN cells, with the same trends as observed for the proteins (Fig. 1H). Furthermore, the Cleaved-*CASPASE*-3 protein, *CASPASE*-3 mRNA (Fig. 1C, G, and H), BAX protein (Fig. 1C, E and H) and mRNA, and the BAX/BCL2 ratio (Fig. 1F) were lower in the P4 group compared with KGN cells exposed to P4 + AG. BAX protein and mRNA, Cleaved-CASPASE-3 protein and *CASPASE*-3 mRNA had no significant differences compared with the P4 group and the control cells (Fig. 1C, E, G, and H).

### PAQR7 mediated the anti-apoptotic effect of P4 on human KGN cells

**Fig. 2:**
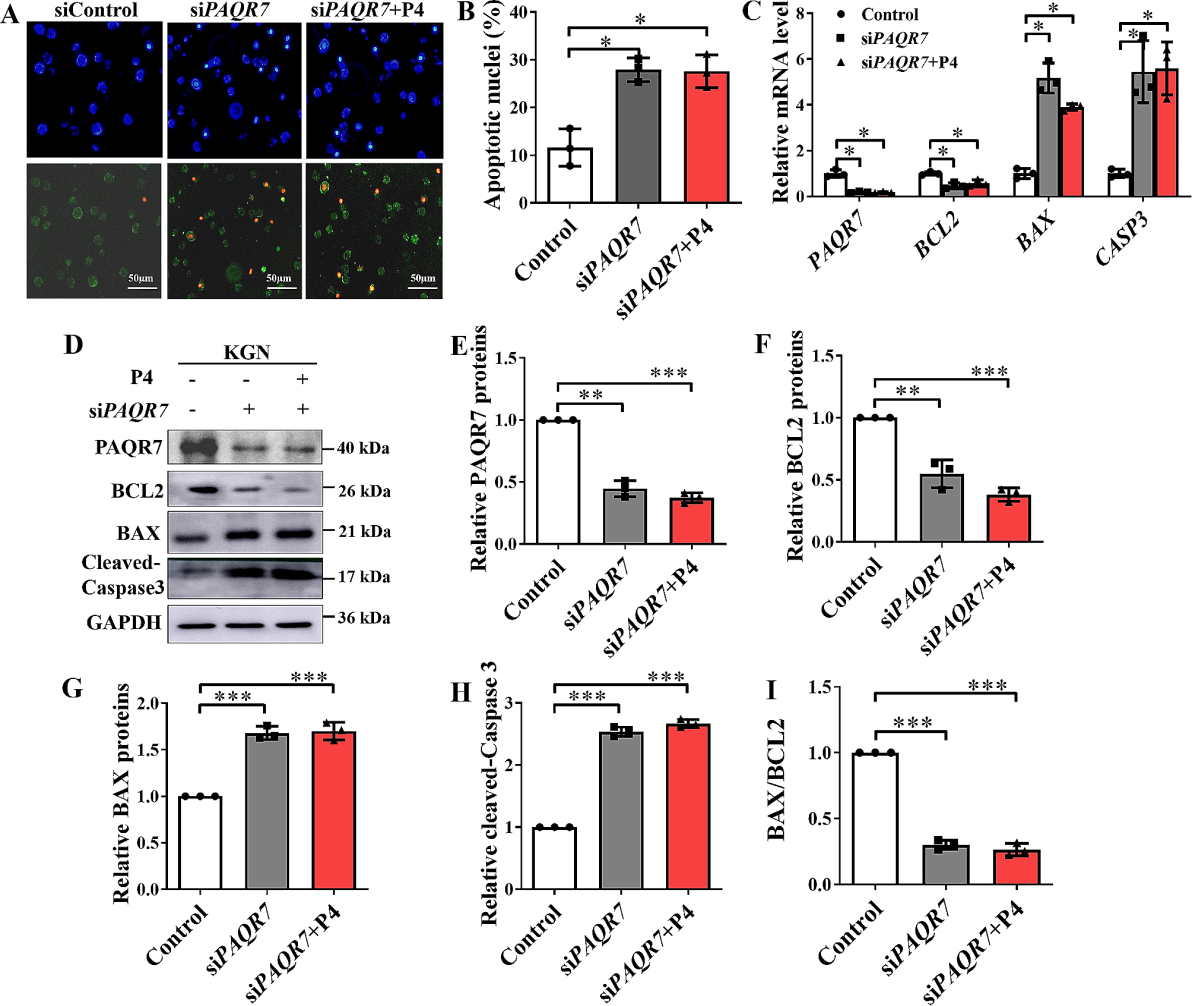
The involvement of PAQR7 in the anti-apoptotic action of progesterone (P4) on KGN cells. (**A** and **B**) The apoptosis of KGN cells transferred with control siRNA (siRNA), si*PAQR*7, and si*PAQR*7 co-incubated with P4 (si*PAQR*7 + P4) was examined after annexin V-Fluorescein 5-isothiocyanate/propidium iodide staining. 4′,6-diamidino-2-phenylindole (DAPI, blue) was used to stain the nuclei. Scale bars represent 50 μm. (**C**) The mRNA levels of *PAQR7* and apoptosis-related genes (*CASPASE*-3, *BAX* and *BCL2*) in KGN cells transfected with control siRNA (siRNA), si*PAQR*7 and si*PAQR*7 co-incubated with P4 (si*PAQR*7 + P4) were analyzed by real-time quantitative PCR. (**D**) A sample of western blot analysis measuring the protein levels of PAQR7, Cleaved-CASPASE-3, BAX, and BCL2. (**E**-**I**) Statistical analyses of PAQR7 (**E**), BCL2 (**F**), BAX (**G**), Cleaved- CASPASE-3 (**H**) and BAX/BCL2 ratio (**I**) were conducted by quantifying the gray value of target bands normalized to that detected by the anti-GAPDH antibody (loading control) using Image J software. Values are presented as the mean ± SEM. The experiment was repeated three times. *P < 0.05, **P < 0.01, ***P < 0.001 compared with the control group, One-way ANOVA analysis

**Fig. 3:**
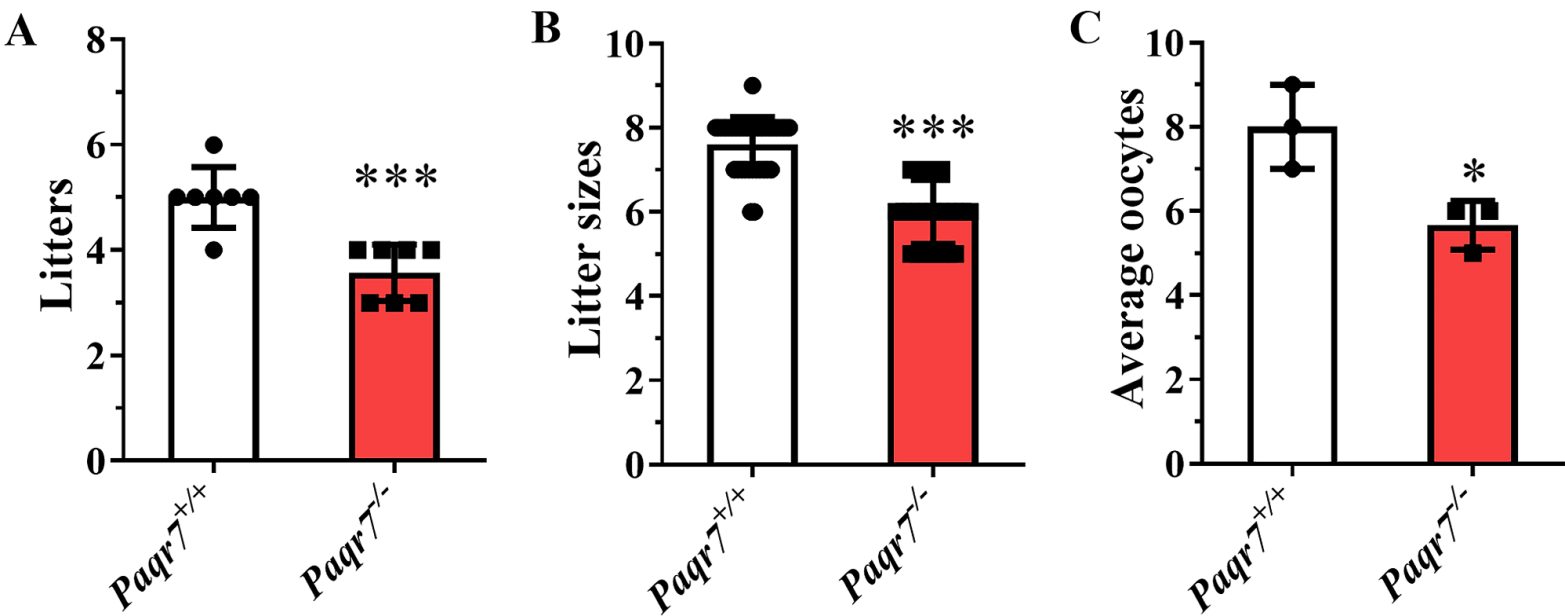
Effect of *Paqr7*-knockout on female fertility. (**A**, **B**) The fertility of *Paqr7*^*−/−*^ female mice was evaluated by being caged with for 6 months and the offsprings were observed and counted regularly. Effects of the *Paqr7* knockout on female fertility. The mating was performed as follows: Wild-type (*Paqr7*^*+/+*^) and *Paqr7*^*−/−*^ female mice were mated with male mice of known fecundity (one male mouse and one female mouse in each cage and seven cages for each group). The litters (**A**) and litter sizes (**B**) were recorded for about six months. (**C**) Statistics on the number of oocytes in the natural ovulation experiment in wild-type female mice (*n* = 3) and *Paqr7*^−/−^ female mice (*n* = 3). Values are presented as the mean ± SD, **P < 0.01, ***P < 0.001, compared with *Paqr7*^+/+^ mice by the student *t*-test

To investigate whether PAQR7 mediated the anti-apoptotic effect of P4 on KGN cells, KGN cells were transfected with control siRNA (siRNA), si*PAQR*7 and si*PAQR*7 co-incubated with P4 (si*PAQR*7 + P4), then examined after annexin V-Fluorescein 5-isothiocyanate/propidium iodide staining, respectively. In the si*PAQR*7 group, both early apoptosis (green in Fig. 2A) and late apoptosis (red in Fig. 2A) of KGN cells increased by about 100% (Fig. 2B), while the expression of *PAQR7* in KGN cells was downregulated by about 70% after transfection with si*PAQR*7 (Fig. 2C–E). The CASPASE-3 mRNA and protein levels of cleaved- CASPASE-3 (Fig. 2C, D and H) and BAX (Fig. 3C, D and G) were upregulated, the mRNA and protein levels of BCL2 (Fig. 2C, D and F) were downregulated, and the BAX/BCL2 ratio (Fig. 2I) was elevated in KGN cells with *PAQR7* knockdown. Compared to the si*PAQR7* group, the exogenous addition of P4 with si*PAQR7* had no effect on apoptosis (Fig. 2A and B), the expression of PAQR7 (Fig. 2D and E), CASPASE-3 (Fig. 3D and H), BAX (Fig. 2Dand G), and BCL2 (Fig. 2D and F), or the BAX/BCL2 ratio (Fig. 3I) in KGN cells.

### Knockout of PAQR7 affected female fertility in mice

To study the role of PAQR7 in female reproduction, we constructed a *Paqr7*^−/−^ mouse model using the CRISPR-Cas9 method. The deletion of 812 bp located in the third exon of *Paqr7* in the *Paqr7*^−/−^ mice resulted in a frameshift mutation in the transcript (Supplementary Fig. S2A). Consequently, translation was terminated prematurely, eliminating amino acids 38–345 (Supplementary Fig. S2A). The *Paqr7*^−/−^ mice were identified by PCR (Supplementary Fig. S2B and C). The mRNA and protein were not detected in the ovaries of *Paqr7*^−/−^ female mice, indicating that the *Paqr7* was successfully knocked out (Supplementary Fig. S2C and E).

Knockout of *Paqr7* did not affect the growth, development, or viability of the female mice, but the fertility of the female mice was reduced. The litters and litter sizes of *Paqr7*^−/−^ female mice were significantly decreased compared with *Paqr7*^+/+^ female mice (*P* < 0.001, Fig. 3A and B). We next went on to examine spontaneous ovulation by the cage overnight, and our result found that the *Paqr7*^−/−^ female mice produced significantly fewer oocytes compared to the wild-type mice (*P* < 0.01, Fig. 3C).

### Knockout of PAQR7 caused abnormal ovarian microstructure and follicle development

**Fig. 4:**
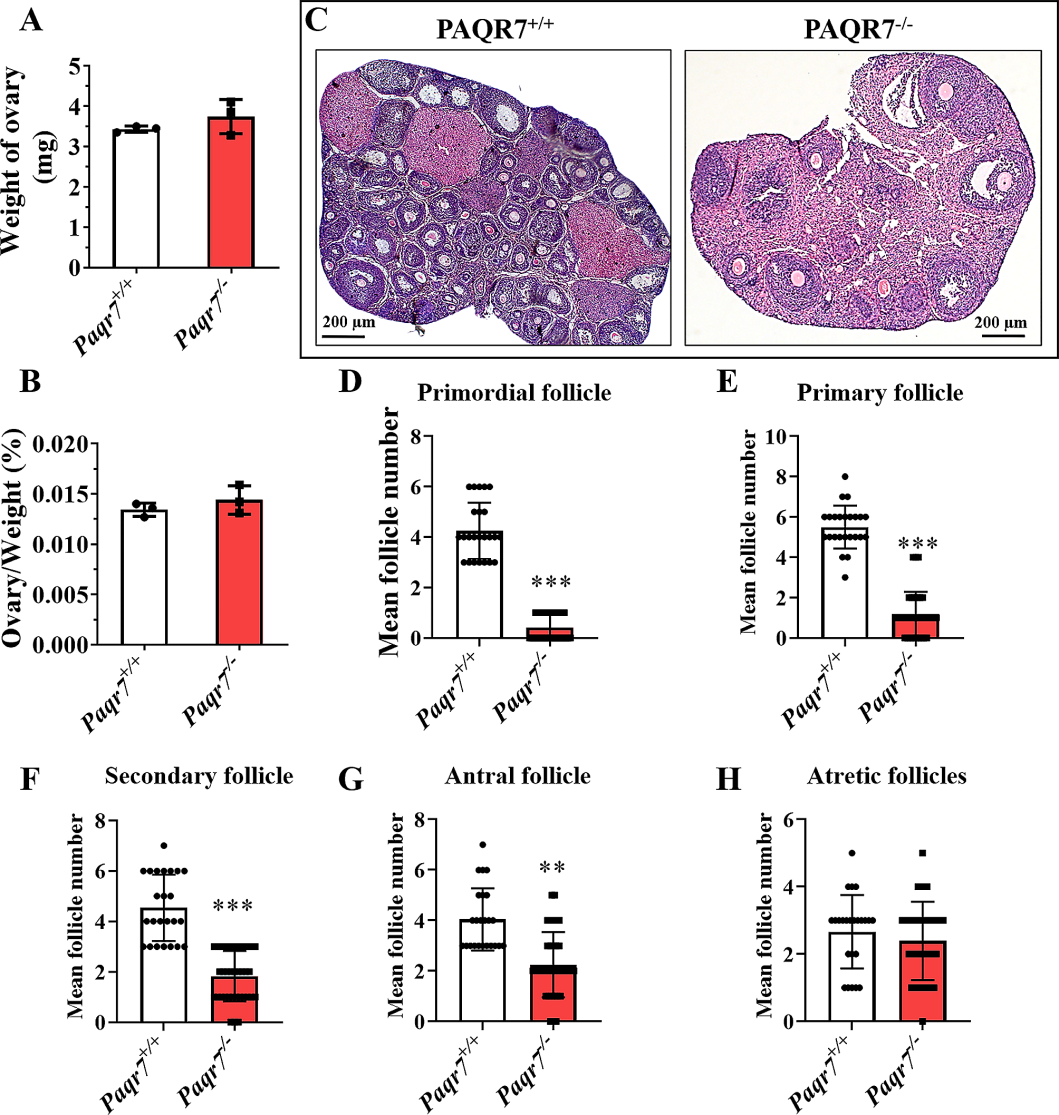
Effect of *Paqr7*-knockout on ovarian weight, microstructure and follicle development. (**A**, **B**) Effects of the *Paqr7* knockout on the weight of ovary and the ovary index. (**C**) The morphologies of ovaries in 2-month-old wildtype (*Paqr7*^+/+^) and *Paqr7*-knockout (*Paqr7*^−/−^) female mice were examined after haematoxylin-eosin (HE) staining. (**D-H**) Statistical analyses of different stages of follicles in 3-month-old *Paqr7*^+/+^ (*n* = 3) and *Paqr7*^−/−^ female mice (*n* = 3) were performed according to the HE staining results. The primary follicles (PF) containing one to two layers of cuboidal granulosa cells, enlarged oocytes, and measuring between 30 and 70 μm in diameter, and final follicle growth and maturation were evaluated by counting the number of mid- and large-antral follicles (ANF), measuring from 250 to > 400 μm. *P < 0.05, compared with *Paqr7*^+/+^ mice by the student *t*-test

The ovary weight and ovary index in *Paqr7*^*−/−*^ female mice were not significantly different from those in wild-type female mice (Fig. 4A and B). However, the histological analysis of ovaries demonstrated that *Paqr7*^*−/−*^ female mice had abnormal ovarian microstructures with ovarian atrophy, cortical thickening, and fewer growth follicles and blood vessels, and the arrangement of luteal granulosa cells was loose and irregular (Fig. 4C). In addition, there were fewer primordial (PMF), primary follicles (PF), secondary follicles (SF) and antral (ANF) follicles in the ovaries of *Paqr7*^*−/−*^ compared with wild-type female mice (Fig. 4C-G), while atretic follicles (ATF) had no difference between *Paqr7*^*−/−*^ and wild-type female mice (Fig. 4C and H).

### Knockout of PAQR7 disturbed female reproductive endocrine

**Fig. 5:**
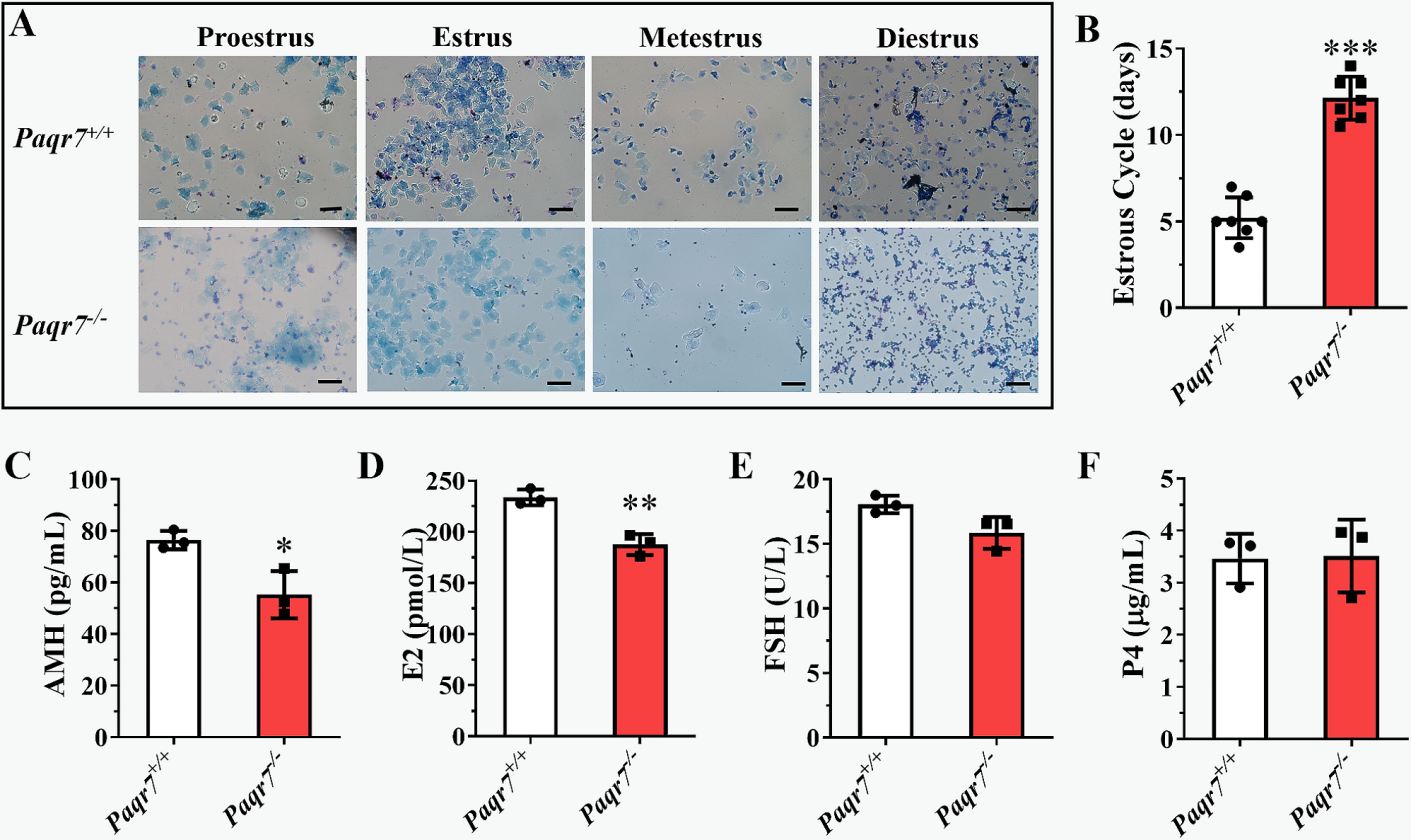
Effect of *Paqr7* knockout on reproductive endocrine function in female mice. (**A**) Analysis of the estrous cycle by smear of exudated cells from the mouse vagina. (**B**) Effects of estrous cycle regularity in *Paqr7*^−/−^ compared with *Paqr7*^+/+^ female mice. *n* = 7, values are presented as the mean ± SEM. ***P < 0.001, compared with *Paqr7*^+/+^ mice by the Student *t*-test. (**C-F**) The concentrations of serum anti-müllerian hormone (AMH, **C**), estradiol (E2, **D**), follicle-stimulating hormone (FSH, **E**), and progesterone (P4, **F**) in 2-month-old *Paqr7*^+/+^ (*n* = 3) and *Paqr7*^−/−^ (*n* = 3) female mice were examined by enzyme-linked immunosorbent assay. Values are presented as the mean ± SEM. *P < 0.05, **P < 0.01, compared with *PAQR7*^+/+^ mice by the student *t*-test

The estrous cycle and the serological indicators of anti-Müllerian hormone (AMH), estradiol (E2), progesterone (P4), and follicle-stimulating hormone (FSH) were examined to assess female reproductive endocrine function. The *Paqr7*^*−/−*^ female mice showed a disturbed and prolonged estrous cycle, and then a large number of white blood cells were appeared in the diestrus period (Fig. 5A and B). Furthermore, *Paqr7*^*−/−*^ female mice had significantly lower levels of serum AMH (*P* < 0.01, Fig. 5C) and E2 (*P* < 0.05, Fig. 5D) than *Paqr7*^*+/+*^ female mice, while there were no differences in serum P4 (Fig. 5E) and FSH between *Paqr7*^*+/+*^ and *Paqr7*^*−/−*^ female mice (Fig. 5F).

### Elevated cell apoptosis in the ovaries of Paqr7^−/−^ female mice

**Fig. 6:**
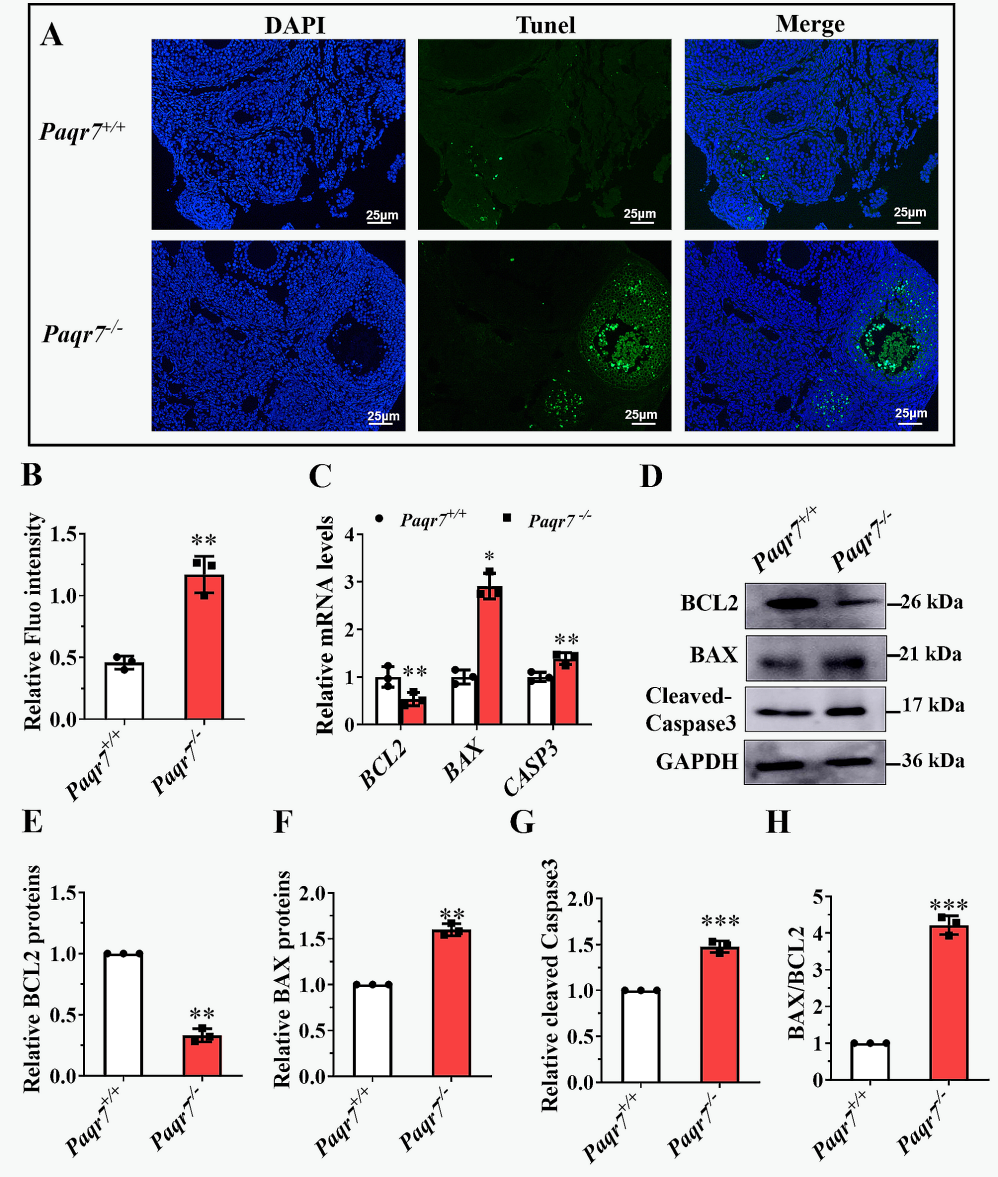
Changes in apoptosis and apoptosis-related factors in the ovaries of *Paqr7*^−/−^ female mice. (**A**) Apoptosis was assessed by TUNEL assay in the ovaries of *Paqr7*^+/+^ (*n* = 3) and *Paqr7*^−/−^ (*n* = 3) female mice. 4′,6-diamidino-2-phenylindole (DAPI, blue) was used to stain the nuclei. Scale bars represent 25 μm. (**B**)The number of apoptotic cells per section in *Paqr7*^+/+^ (*n* = 3) and *Paqr7*^−/−^ (*n* = 3) female mouse ovaries. (**C**) The mRNA levels of *Bcl2*, *Bax* and *Caspase-3* were examined in the ovaries of *Paqr7*^+/+^ (*n* = 3) and *Paqr7*^−/−^ female mice (*n* = 3) by real-time quantitative PCR. (**D**) A sample of Western blot analysis measuring the protein levels of Bcl2, Bax, and Cleaved-Caspase-3. (**E**-**H**) Statistical analyses of the protein levels of Bcl2 (**E**), Bax (**F**), Cleaved-Caspase-3 (**G**), and Bax/Bcl2 ratio (**H**) in the ovaries of *Paqr7*^+/+^ (*n* = 3) and *Paqr7*^−/−^ female mice (*n* = 3) were conducted by quantifying the gray value of target bands normalized to that detected by anti-GAPDH antibody (loading control) using Image J software. Values are presented as the mean ± SEM. *P < 0.05, **P < 0.01, ***P < 0.001 compared with the *Paqr7*^+/+^ female mice by the student *t*-test

The TUNEL assay revealed that the number of TUNEL-positive cells increased significantly in the ovaries of *Paqr7*^*−/−*^ compared with *Paqr7*^*+/+*^ female mice (Fig. 6A and B). Meanwhile, *Bcl2* mRNA was significantly decreased (*P* < 0.01), while *Bax* and *Casp3* mRNAs were significantly increased (*P* < 0.05) in the ovaries of *Paqr7*^*−/−*^ compared with *Paqr7*^*+/+*^ female mice (Fig. 6C). The Western blot assay showed that Bcl2 (Fig. 6D and E) and Bax (Fig. 5D and F) proteins demonstrated similar changes to their mRNA levels, and the the Bax/Bcl2 ratio (Fig. 5H) and cleaved -Caspase3 (Fig. 5D and G) were elevated in the ovaries of *PAQR7*^*−/−*^ compared with *Paqr7*^*+/+*^ female mice. In addition, ROS were elevated within the ovaries of *Paqr7*^*−/−*^ female mice (Supplementary Fig. S3).

### PAQR7 protein was downregulated in the GCs of DOR-patients and the ovaries of mice with a DOR-like phenotype

**Fig. 7:**
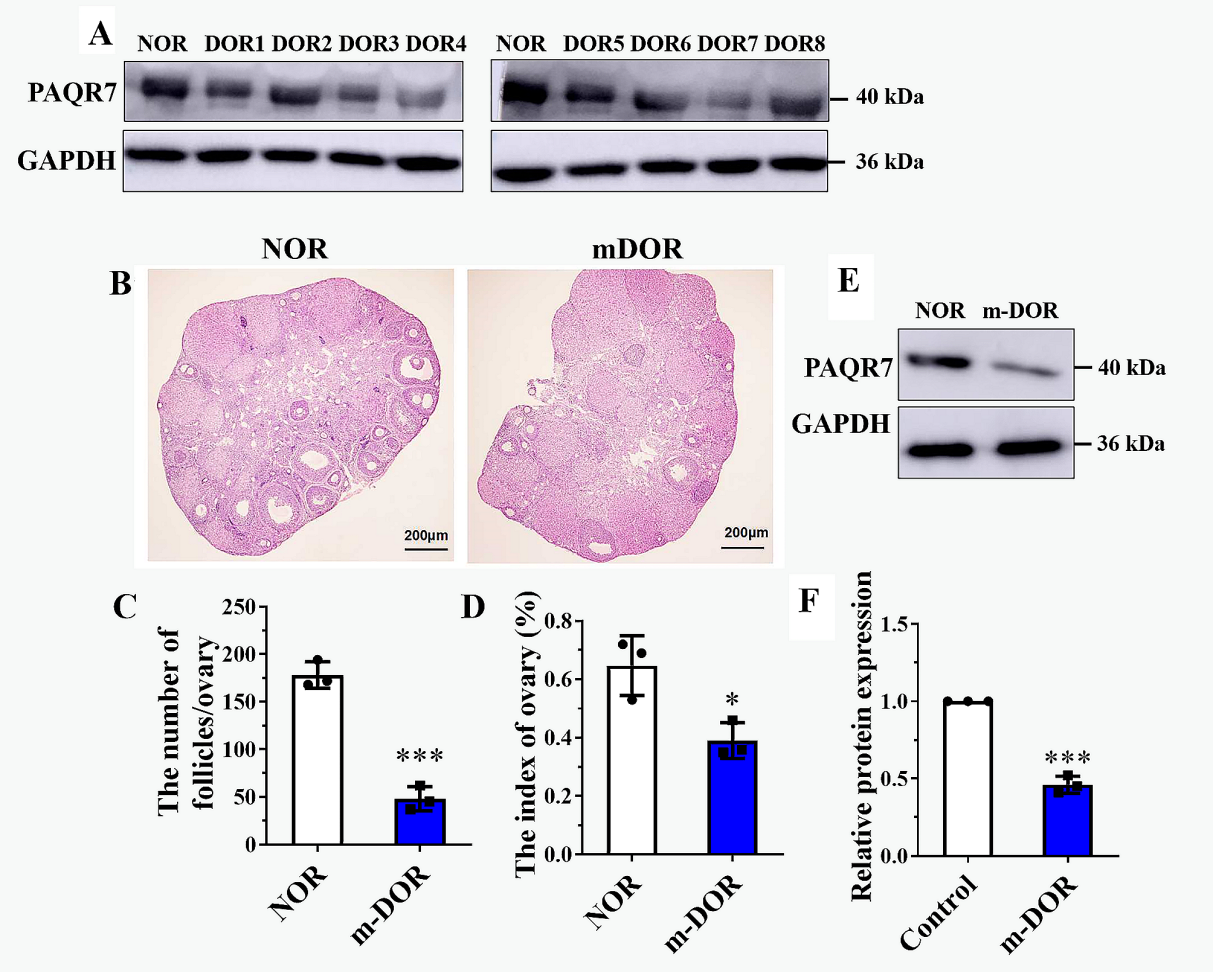
The protein levels of PAQR7 in DOR patients and mice. (**A**) The protein levels of PAQR7 were examined in the granulosa cells (GCs) isolated from female suffering from patients diagnosed with diminished ovarian reserve (DOR, *n* = 8) and the Control group (*n* = 11). (**B**) Construction of the mouse model for decreased ovarian reserve (DOR). Assessment of ovarian follicles via HE-staining assays in DOR mice ovaries. Scale bars represent 200 μm. (**C**) The numbers of follicles/ovary were reduced in DOR mice. (**D**) The ovarian index in DOR mice. (**E, F**) The protein level of PAQR7 in the ovary of DOR mice (*n* = 3) and the control group (*n* = 3). *P < 0.05, ***P < 0.001, compared with the control mice by the student *t*-test

Since the knockout of *Paqr7* caused ovarian dysfunction in mice, we evaluated whether the expression of PAQR7 was related to DOR, a common disease of ovarian dysfunction. Western blot assay showed a lower level of PAQR7 protein in GCs isolated from the follicular fluids of patients with DOR (Fig. 7A). Meanwhile, we also constructed the mouse model for decreased ovarian reserve (DOR, Fig. 7B-D), and found that the ovaries of mice with a DOR-like phenotype had significantly lower PAQR7 levels in mice when compared to the control group (*P* < 0.001, Fig. 7E, F).

## Discussion

Normal proliferation and apoptosis of GCs play decisive roles in promoting follicular initiation, development, and maintenance of ovarian function, while abnormally increased apoptosis of GCs is associated with follicular atresia [5, 40, 41], endocrine disorders, and ovarian aging and reserve [1, 4]. It has been known for over three decades that P4 prevents the apoptosis of ovarian granulosa cells (GCs), which do not express the classic nuclear P4 receptor [42]. As a member of the progestin and adipoQ receptor superfamily, PAQR7 (mPRα) has the typical P4-binding characteristics of P4 membrane receptors [43]. Although there have been many fish studies describing how PAQR7 mediates nongenomic responses of P4 [21, 22], whether it mediates P4 actions on GCs in mammals remains unclear. In this study, we tried to determine whether PAQR7 mediated the anti-apoptotic effects of P4 in human KGN cells and the effect of its deficiency on ovarian function in female mice *Paqr7* knockout. Our data provided novel insights that PAQR7 is involved in the inhibitory effect of P4 on apoptosis in human GCs and is an important protein in maintaining ovarian function in female mice.

The Bcl2 family of proteins and Caspase-3 are involved in regulating cell life and death [44, 45]. Previous studies have shown that apoptosis of GCs is regulated by caspase-3 and Bcl2 gene family members, including Bax and Bcl2 [40, 46]. We found that these apoptosis-related proteins are also involved in P4–PAQR7 signaling–mediated anti-apoptotic actions, indicating that P4–PAQR7 may regulate apoptosis of KGN cells through CASPASE-3 and BCL2 gene family members. These results are consistent with a previous study that showed the PGRMC1 is involved in mediating P4 to maintain the survival of KGN cells by suppressing BAX and increasing BCL2 expression, thereby changing the BAX/BCL-2 ratio to favor cell survival [25]. In addition, evidence from fish models has shown that PAQR7 couples to inhibitory G protein or olfactory G protein to regulate membrane-bound adenylyl cyclase activity and initiate downstream signaling pathways such as PI3K/Akt and MAPK p42/44 pathways [20, 47, 48]. Hence, it is interesting to further our study to determine whether PAQR7 couples to these proteins and regulates the downstream signaling to mediate the anti-apoptotic action of P4 on GCs.

Decreased ovarian function directly leads to abnormal secretion of human sex hormones and follicle development [4]. Compared with the wild-type female mice, the *Paqr7* knockout female mice had a decreased serum concentration of E2. AMH was mainly expressed in GCs of ANF and 4 mm-diameter small antral follicles and not in ATF. AMH level is a sensitivity indicator of ovarian dysfunction, which can be used as a marker for reflecting ovarian reserve [49]. In this study, we detected that serum AMH levels markedly decreased in *Paqr7* knockout female mice compared with the wild-type female mice. Furthermore, the level of FSH showed that there were no significant changes in *Paqr7* knockout mice and wild-type mice, which reflects that the hypothalamus vertical excitation element has no obvious difference between wild-type mice and *Paqr7* knockout mice. These results also proved once again that the absence of PAQR7 has adverse effects on ovarian deficiency.

Niswender et al. have demonstrated that the corpus luteum (CL) is a temporary endocrine gland that has a critical role in the establishment and maintenance of pregnancy [50]. During the life span of the corpus luteum, this transient reproductive gland undergoes several physiological events, including growth, function, and regression [51]. Interestingly, the results of HE staining showed that there were significant differences in the structure of the corpus luteum of the ovary, that is, the corpus luteum of *Paqr7* knockout female mice had fragmentation, fibrosis, and other phenomena, and the deletion of the PAQR7 gene resulted in a significantly higher amount of this abnormal form of corpus luteum. Furthermore, the fertility rate and native ovulation would indicate that there were major changes in luteal function, as developing embryos were able to implant and mature to full-term. In addition, *Paqr7* knockout female mice still observed that estrous cycles, with the only difference being a prolonged estrus, which further supports the abnormities of ovarian function in *Paqr7* knockout mice. As we all know, the Star, Cyplla1, 3β-Hsd1and Bhmt have been found to play an important role in maintaining the function and morphology of the corpus luteum [52, 53]. Thus, further studies are needed to determine if a deletion of related genes could lead to a more prominent luteinization of *Paqr7* knockout mice.

In addition, PMF are honored as the “warehouse” of ovarian function. Continuous and orderly activation of PMF is the essential for maintaining ovarian function, and excessive activation or abnormal atresia of the follicle can accelerate ovarian aging [34]. Antral follicle count is one of the important indicators of fertility, and the higher the number of ANF, the higher the chance of conception. Here, fewer PMF and ANF were observed in the ovaries of *PAQR7* knockout female mice than those in wild-type female mice, indicating that the knockout of *Paqr7* may affect ovarian reserve function and real-time function in mice. Meanwhile, the molecular level detection showed that knockout of *Paqr7* induced an increase in overall ovarian apoptosis, enhanced activation of the primordial follicle activation pathway, and aggravated oxidative stress damage (Figure S3). Furthermore, the TUNEL results showed that, compared with the ovary of wild-type mice, the ovary of 2-month-old *Paqr7* knockout mice showed many obvious apoptotic signals, indicating that the decrease in growing follicles may be caused by apoptotic atresia. In vitro experimental verification results also showed a consistent pattern (Fig. 2). Therefore, we conclude that the knockout of *Paqr7* in mice reproduced some of the key ovarian dysfunction features, including elevated apoptosis in the ovaries, follicular development failure, and reduced female fertility. However, as complete sterility was not observed in the *Paqr7* knockout female mice, it is plausible to consider the existence of compensatory mechanisms regulating gene expression within its receptor family. Notably, no significant differences were found in the expression levels of *PGRMC1*, *PGRMC2*, and *CYB5D2* between *Paqr7* knockout and wild-type female mice (Figure S4). Conversely, a significant upregulation was observed in the expression levels of *Pgr*, *Nenf*, *Paqr5*, *Paqr6*, *Paqr8* and *Paqr9* genes (Figure S4, *P* < 0.05). These findings suggest that certain members within the receptor family may compensate for and assume some physiological functions of PAQR7 to ultimately manifest the fertility phenotype observed in female mice lacking this protein. Therefore, additional investigations are warranted to elucidate whether these receptor types truly complement each other in function.

**Fig. 8:**
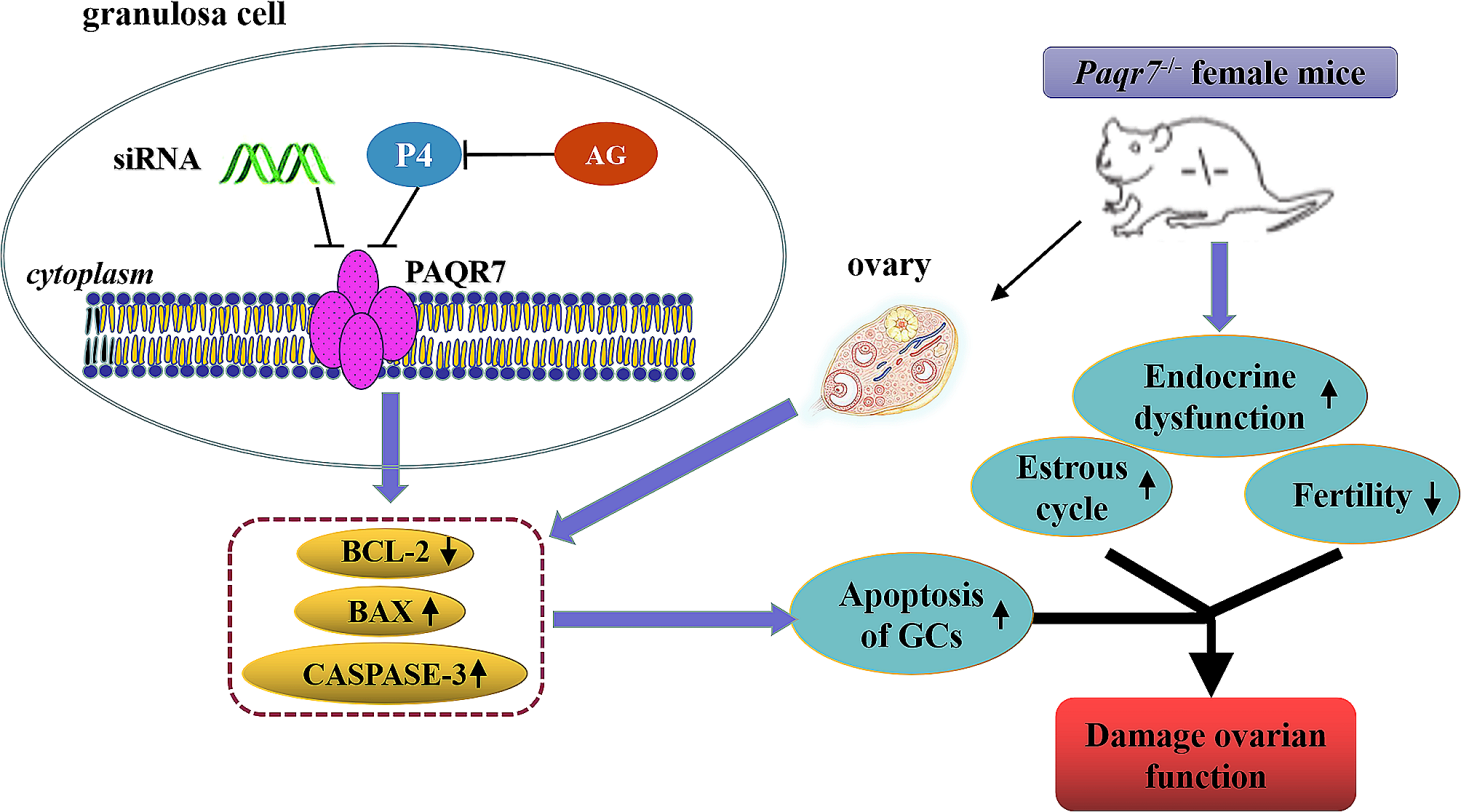
Working model. A novel molecular network controlling GC apoptosis composed of PAQR7 and the BCL-2/BAX/CASPASE-3 signaling pathway. Knockout of the *Paqr7* gene in female mice could reduce ovarian function

To further ensure the correlation between PAQR7 and ovarian function decline, we used the follicular fluids of patients with DOR and found that downregulated expression of PAQR7 was detected in GCs isolated from follicular fluids of patients with DOR compare with the control group. More importantly, the lower levels of PAQR7 were also found in DOR of mice than those in the normal controls. These results suggest that the PAQR7 plays a crucial role in the occurrence of DOR. In addition, our data also provide a potential therapeutic method for delaying the onset of ovarian aging, clinical treatment of POF, female infertility and other diseases. In summary, this study has shown the physiological role of PAQR7, specifically in ovarian function, and the anti-apoptotic action of P4. PAQR7 deficiency aggravates apoptosis of KGN cells via the BCL-2/BAX/CASPASE-3 signaling pathway. The deletion of *Paqr7* in female mice activated GCs apoptosis to damage ovarian function (Fig. 8). These findings shed new light on the underlying mechanism of the anti-apoptotic action of P4 on GCs and the roles of PAQR7 in female reproduction in mammals.

### Electronic supplementary material

Below is the link to the electronic supplementary material.

Supplementary Material 1: Supplementary figures and tables

## Data Availability

Data sharing is not applicable to this article.
