# Supplementary material for: Progestin and adipoQ receptor 7 (PAQR7) mediate the anti-apoptotic effect of P4 on human granulosa cells and its deficiency reduces ovarian function in female mice
Source: J Ovarian Res. 2024 Feb 6;17:35. doi: 10.1186/s13048-024-01348-w (PMC10845654; doi:10.1186/s13048-024-01348-w)
Supplement: Supplementary file 1 — Supplementary Material 1: Supplementary figures and tables [file 13048_2024_1348_MOESM1_ESM.doc]

**Supplementary figures and tables**


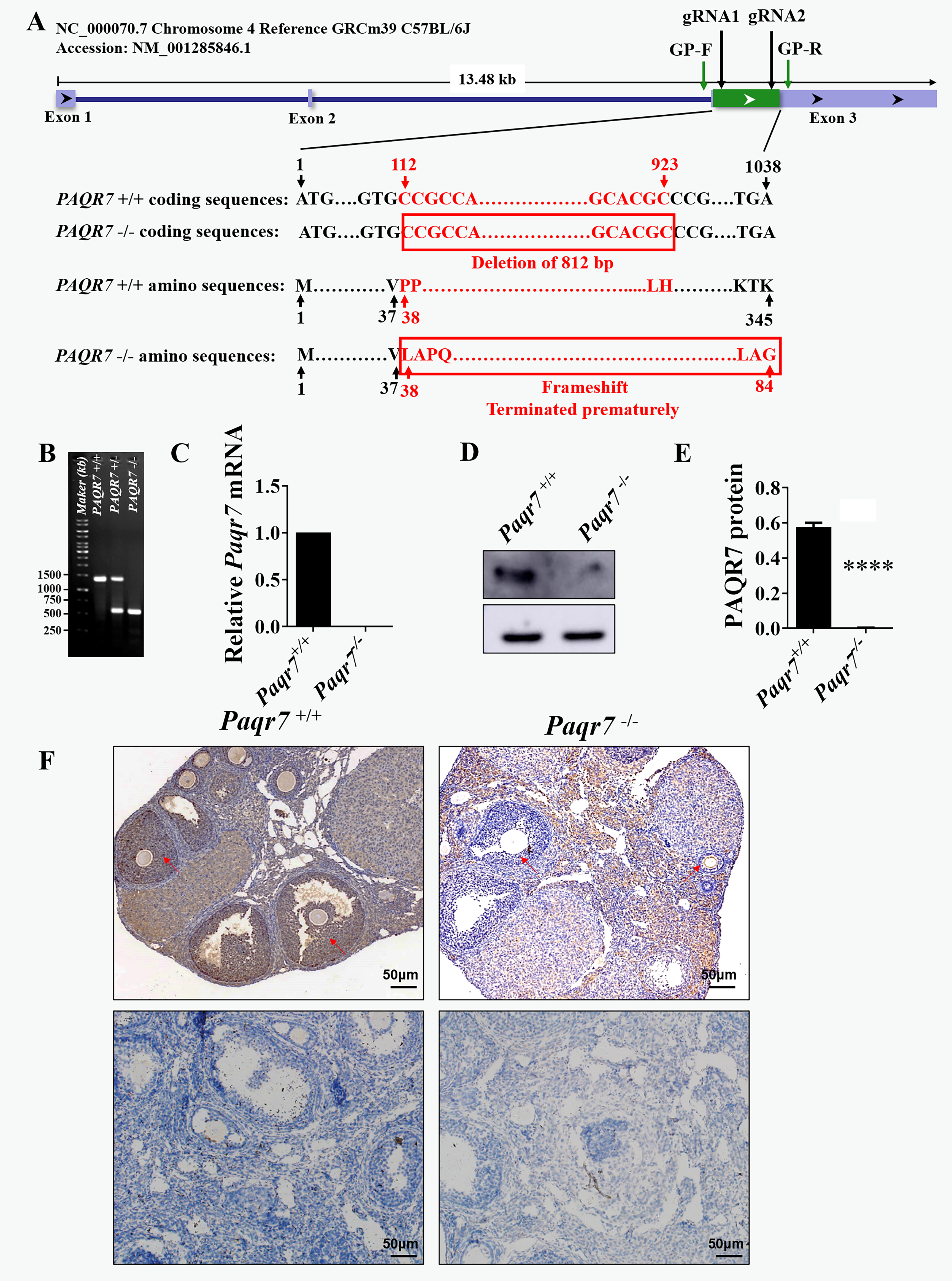


**Figure.S1 Construction and characterization of PAQR7 gene knockout females**. (**A**) Construction and characterization of PAQR7 gene knockout females. The knockout region is CCGCCA in the third exon of PAQR7. GCACGC has a total of 812 bases, frameshift mutation occurs after the deletion of this segment, and the encoded protein is missing. LHQKTK has 261 amino acids (PAQR7 protein has 345 amino acids). (**B-C**) The identification of F2 offspring in PAQR7^-/-^ female mice by PCR based genotyping. The genomic DNA of mouse tail was obtained through genotype identification, and then PCR reaction was performed with designed primers to verify that PAQR7 was successfully knocked out at the DNA level (B); The mRNA expression was detected by fluorescent quantitative PCR (C). (D-E) The expression changes of PAQR7 marker during PAQR7^+/+^ and PAQR7^-/-^ mice at 3-months of age by western-blot assay. (F) The representative immunohistochemical (IHC) staining of PAQR7 in mouse ovaries with PAQR7^+/+^ and PAQR7^-/-^ mice (10X). *P < 0.05, ***P < 0.001, compared with PAQR7^+/+^ mice by Student t-test.


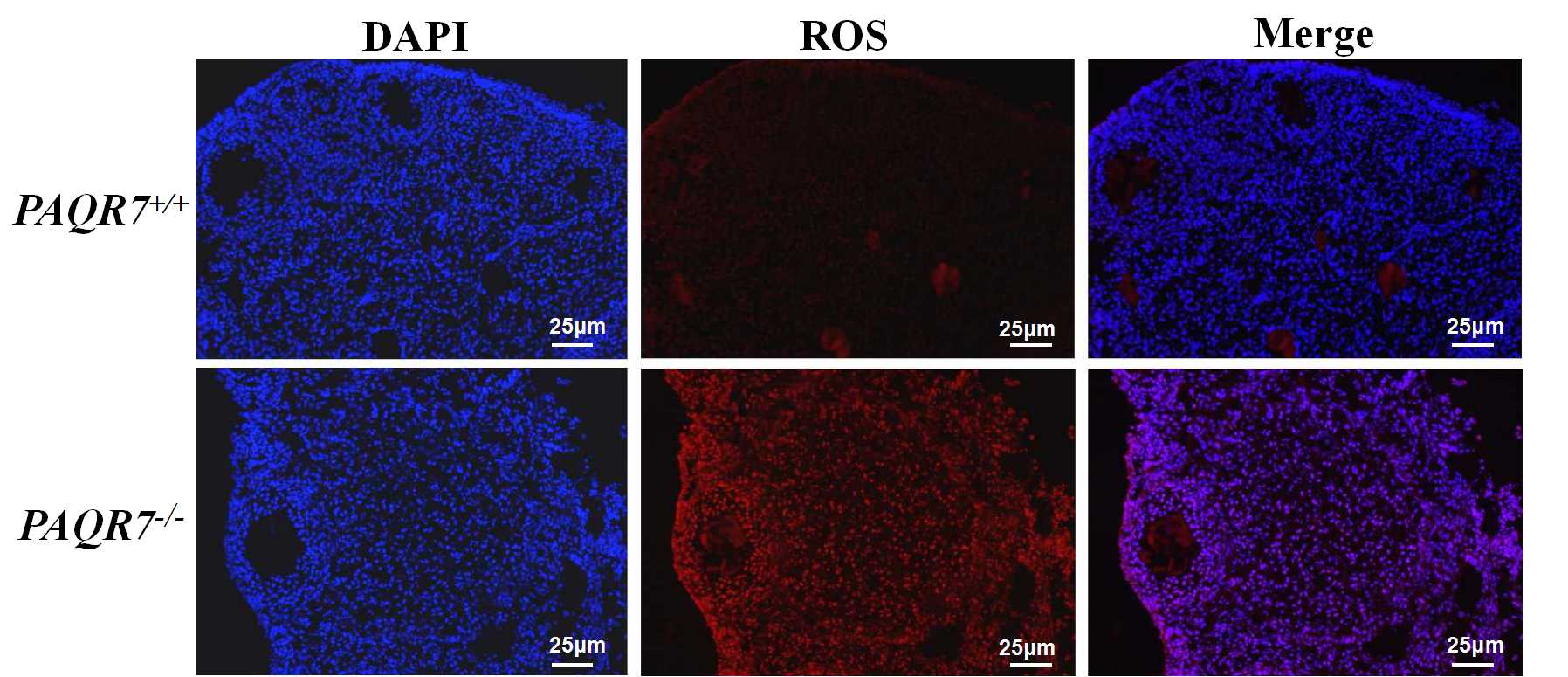


**Figure.S2 The level of ROS was detected by dihydroethidium staining method (Sigma-D7008, USA) in the ovaries of PAQR7^+/+^ and PAQR7^-/-^ female.**


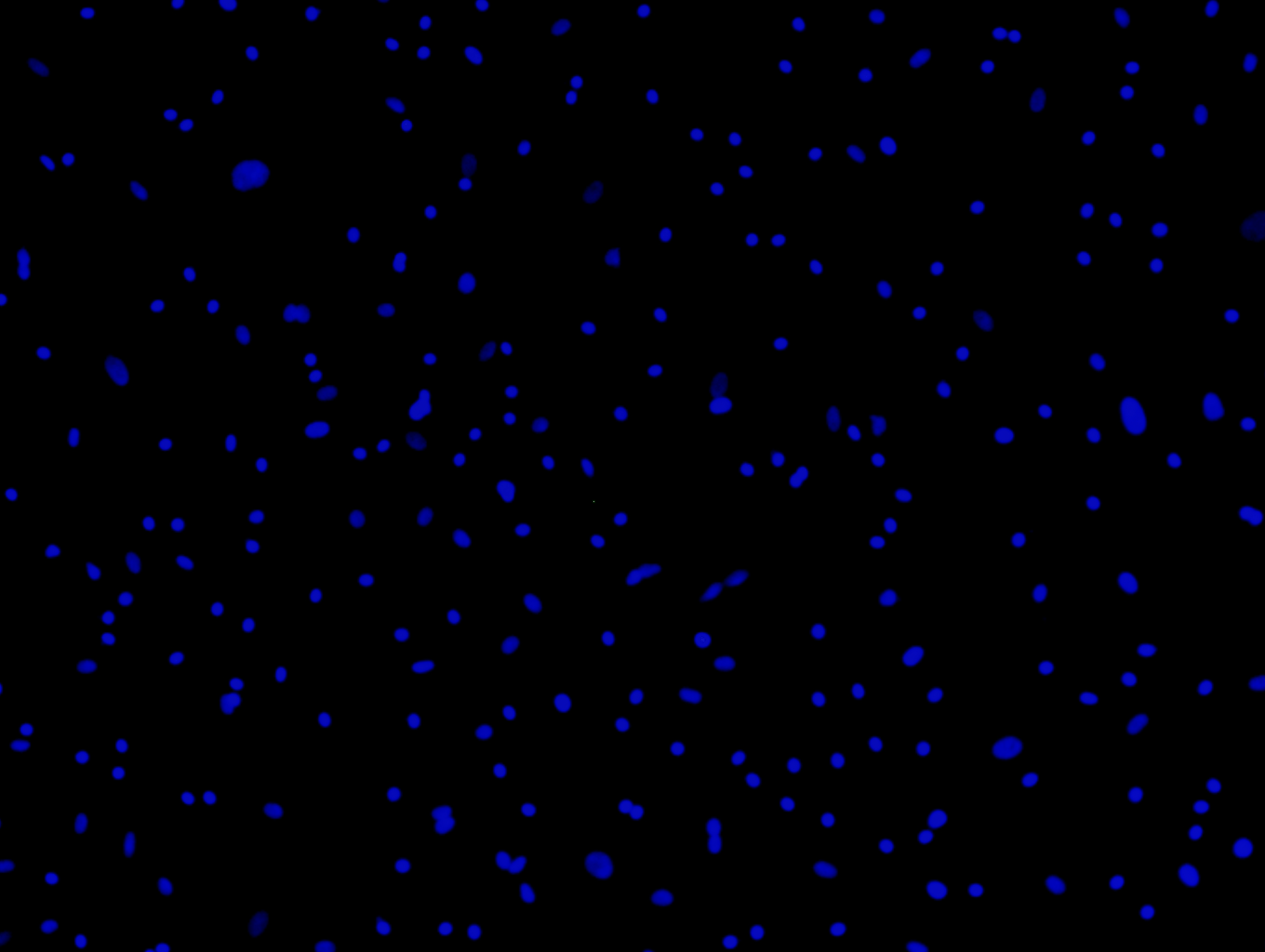

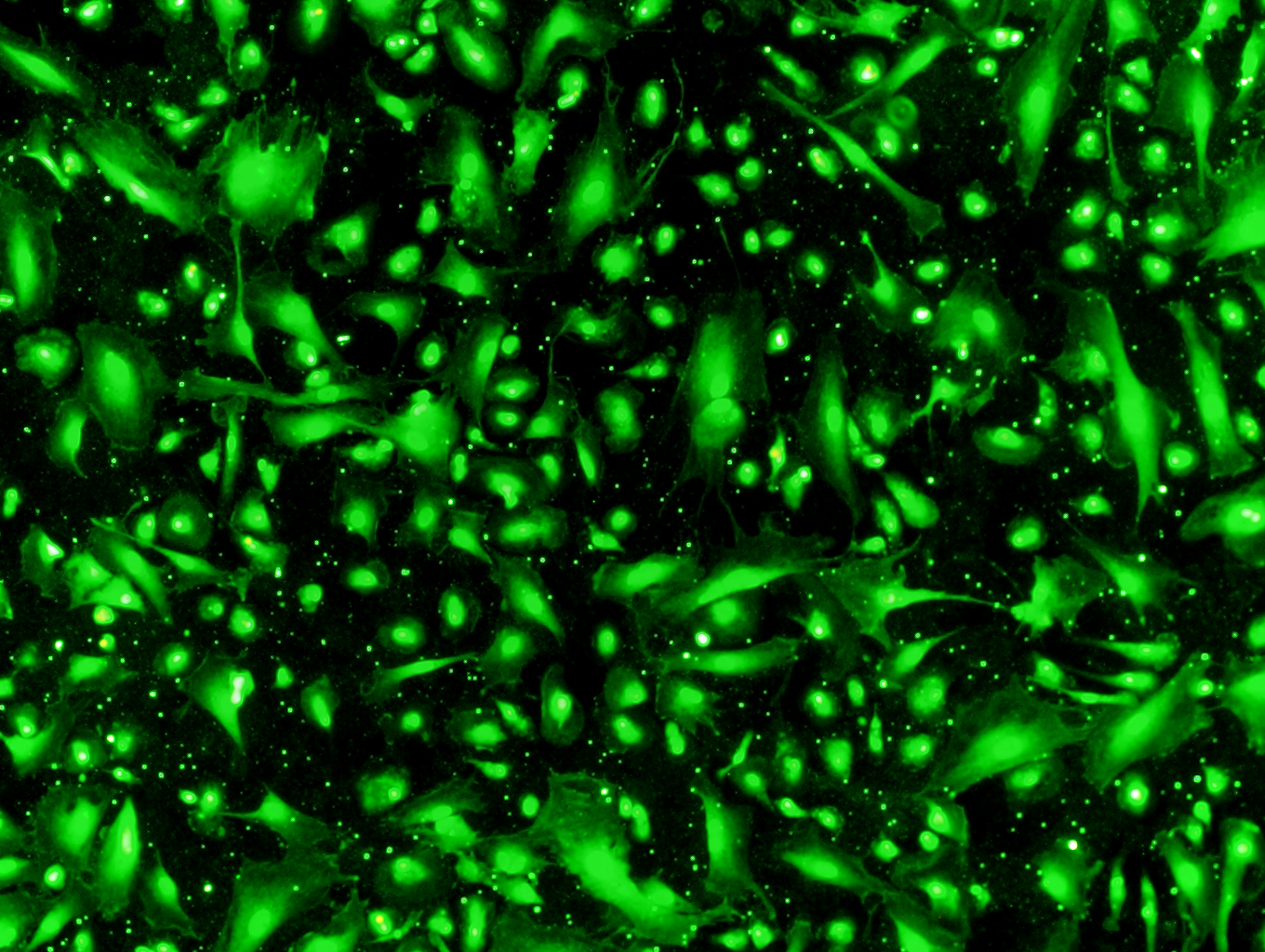


**Figure. S3 Immunohistochemical staining for FSHR for identification of the follicular granulose cells (X10)**

**
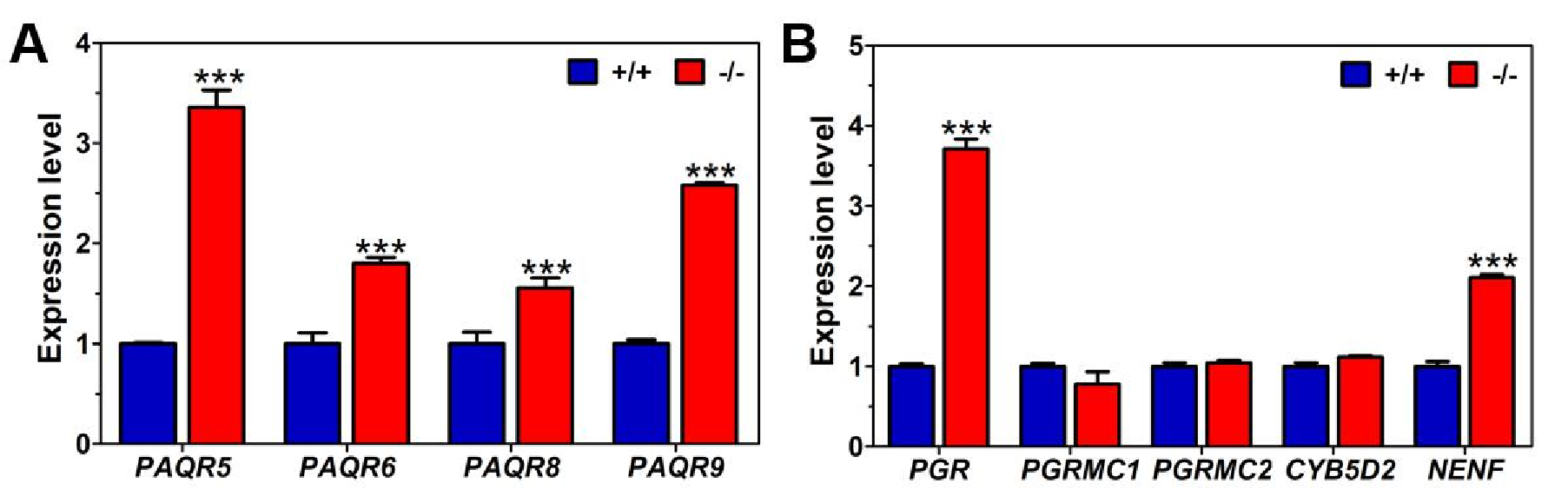
**

**Figure.S4 PAQR7 knockout affects the expression of progesterone receptor family genes. (A) Expression levels of PAQR7 family progesterone receptors in PAQR7 female mice. (B) Table of progesterone nuclear receptor and membrane receptor MAPR family genes up to the level.**

**Table. S1** Eligibility and Exclusion Criteria.

| Basic inclusion criteria |
| --- |
| 1. Older than 18 , less than 35 years of age 2. Sign the informed consent 3. With the super can take to 1 or more oocytes - granular cell complex 4. Did not affect ovarian function after ovarian surgery, radiation and chemotherapy, as well as the processing, no abnormal chromosome |
| The normal ovarian storage function group (NOR) |
| 1. Because of the man factors or infertility caused by tubal factor 2. AMH ≥1.1ng/mL 3. No. of retrieved oocytes ≥5 |
| The decreased ovarian reserve group (DOR) |
| 1. Infertility caused by ovarian function decline 2. AMH<1.1ng/mL 3. No. of retrieved oocytes：1~4 |

**Table. S2** The list of human follicular fluid samples

| DOR |  |  |  |  |  |  |  |
| --- | --- | --- | --- | --- | --- | --- | --- |
| age | pathogenic diagnosis | FSH（mIU/ml） | E2 (pmol/L) | LH(mIU/ml) | P4(nmol/L) | AMH(ng/mL) | No. of  retrieved oocytes |
| 32 | DOR | 6.65 | 10.56 | 2.85 | 1.15 | 0.76 | 3 |
| 25 | DOR | 6.22 | 10 | 0.95 | 0.17 | 0.834 | 3 |
| 31 | DOR | 16.7 | 18 | 5.06 | 0.06 | 0.41 | 3 |
| 28 | DOR | 7.82 | 60.67 | 3.63 | 0.894 | 0.95 | 3 |
| 32 | DOR | 4.92 | 21.36 | 1.11 | 0.5 | 0.51 | 1 |
| 30 | DOR | 17.5 | 5.01 | 1.15 | 0.05 | 0.257 | 2 |
| 31 | DOR | 9.43 | 434 | 1.31 | 0.742 | 0.05 | 1 |
| 28 | DOR | 4.87 | 37 | 1.01 | 0.43 | 0.1 | 1 |
| NOR |  |  |  |  |  |  |  |
| age | pathogenic diagnosis | FSH（mIU/ml） | E2 (pmol/L) | LH(mIU/ml) | P4(nmol/L) | AMH(ng/mL) | No. of  retrieved oocytes |
| 32 | endometritis | 3.11 | 111 | 5.82 | 8.33 | 4.23 | 11 |
| 21 | history of Cesarean section | 4.92 | 31.4 | 2.53 | 3.84 | 2.1 | 9 |
| 33 | Abnormal Child-Bearing History | 6.34 | 28.04 | 6.25 | 1.6 | 2.38 | 15 |
| 29 | Uterus scar | 6.37 | 28.8 | 4.44 | 4.96 | 7.1 | 16 |
| 34 | history of Cesarean section | 7.42 | 51.73 | 6.17 | 4.78 | 1.94 | 10 |
| 31 | history of Cesarean section | 5.98 | 76.5 | 1.92 | 4.39 | 3.36 | 16 |
| 34 | endometritis | 4.95 | 81.2 | 5.05 | 7.25 | 3.8 | 17 |
| 35 | endometritis | 4.22 | 128 | 4.97 | 5.93 | 5.04 | 15 |
| 24 | endometritis | 3.4 | 102 | 14.8 | 7.1 | 4.63 | 19 |
| 30 | endometritis | 8.23 | 50.9 | 6.33 | 8.06 | 1.69 | 10 |
| 30 | endometritis | 6.72 | 21.3 | 3.19 | 1.98 | 4.21 | 13 |

(NOR:normal ovarian storage function group; DOR:decreased ovarian function )

**Table S3**. Primers used for quantitative-PCR

| **Gene** | **Primer Sequence(5’-3’)** | **Sequence number** | **Amplified fragment length (bp)** |
| --- | --- | --- | --- |
| PAQR7 | F:GTGTATTATTGTAGCACCAACTCG  R:CACCCTTGTACTATCTGTCGAACT | NM:010029.2 | 143 |
| Bcl2 | F:ATCGCCCTGTGGATGACTGAGT  R:GCCAGGAGAAATCAAACAGAGGC | NM_007445.3 | 137 |
| Bax | F:TCAGGATGCGTCCACCAAGAAG  R:TGTGTCCACGGCGGCAATCATC | NM_001199247 | 124 |
| Caspase3 | F:GCTTGGAACGGTACGCTAAG  R:CCACTGACTTGCTCCCATGT | NM_177821 | 157 |
| GAPDH | F:GGAAGCTTGTCATCAATGGAAATC  R:TGATGACCCTTTTGGCTCCC | NM_002046 | 168 |

**Table.S4. IACUC study protocol**

| **Animal Use Permit** | |
| --- | --- |
| **License No. :** | **SYXK2021-0004** |
| **Name of the entity:** | **Nanchang University** |
| **Legal representative:** | **Changbing Zhou** |
| **Facility Address:** | **Medical Laboratory Animal Center, Nanchang University, No. 71 East Yangming Road, Nanchang city** |
| **Application:** | **barrier environment** |
